# Awareness of and Willingness to Use Oral Pre-Exposure Prophylaxis for HIV Prevention among HIV-Serodiscordant Heterosexual Couples: A Cross-Sectional Survey in Xinjiang, China

**DOI:** 10.1371/journal.pone.0067392

**Published:** 2013-07-16

**Authors:** Peierdun Mijiti, Dilixiati Yahepu, Xiaoni Zhong, Yong Sun, Ting Zhao, Zhen Zhao, Zaiyinuer Abuduaili, Hongfang Zhou, Fanliang Meng, Jianghong Dai, Ailong Huang

**Affiliations:** 1 School of Public Health, Xinjiang Medical University, Urumqi, Xinjiang Uyghur Autonomous Region, China; 2 Department of Disease Control and Prevention, Bureau of Health, Urumqi, Xinjiang Uyghur Autonomous Region, China; 3 School of Public Health, Chongqing Medical University, Chongqing, China; 4 Virus Hepatitis Research Institute, Chongqing Medical University, Chongqing, China; Institut Pasteur of Shanghai, Chinese Academy of Sciences, China

## Abstract

**Objectives:**

We aimed to investigate the awareness of and willingness to use oral pre-exposure prophylaxis (PrEP) for HIV prevention among HIV-negative partners in HIV-serodiscordant heterosexual couples in Xinjiang, China and determine factors that predict willingness to use oral PrEP.

**Methods:**

Between November 2009 and December 2010, a cross-sectional survey was carried out among 351 HIV-negative partners in HIV-serodiscordant heterosexual couples from three cities in Xinjiang, China. Participants completed a self-administered questionnaire to assess their awareness of and willingness to use oral PrEP. Additionally, blood samples were collected to test for HIV infection. Univariate and multivariate logistic regression analyses were performed to identify predictors of willingness to use oral PrEP.

**Results:**

Only 10 participants (2.8%) reported having heard of PrEP, and only two reported ever using PrEP. However, 297 (84.6%) reported that they were willing to use oral PrEP if it was proven to be both safe and effective. Results of multivariate analysis revealed the following independent predictors of willingness to use oral PrEP: monthly household income (adjusted odds ratio = 2.78, <1000 RMB vs. ≥1000 RMB, 95% confidence interval: 1.36–5.69), perceived likelihood of contracting HIV from HIV-positive partner (adjusted odds ratio = 2.63, likely vs. unlikely, 95% confidence interval: 1.12–6.19), and worrying about being discriminated against by others due to oral PrEP use (adjusted odds ratio  = 9.43, No vs. Yes, 95% confidence interval: 3.78–23.50).

**Conclusions:**

Our results showed HIV-negative partners in HIV-serodiscordant heterosexual couples in China had low awareness of oral PrEP but high willingness to use oral PrEP for HIV prevention. Cost of oral PrEP should be taken into consideration in future PrEP prevention strategy. In addition, efforts should be made to reduce stigma attached to oral PrEP use, which may increase its acceptability among potential users.

## Introduction

Thirty years after the first reported case of acquired immune deficiency syndrome (AIDS), prevention of human immunodeficiency virus (HIV) infection remains a public health priority, particularly in low- and middle-income countries. In China, an estimated 780,000 individuals were HIV-positive at the end of 2011, with 48,000 new infections in 2011 [1]. Although the prevalence of HIV in China is relatively low (0.06%), 75.80% of the new infections in 2011 occurred in six provinces: Yunnan, Guangxi, Henan, Sichuan, Xinjiang, and Guangdong. The prevalence of HIV in Xinjiang was 0.17% at the end of 2011; This was the fifth highest rate among Chinese provinces and almost three times that of the national average [2].

In Xinjiang, sexual transmission has been the dominant mode of HIV transmission since 2008. The period of 2010 to 2011 saw a 20% increase in the proportion of new HIV infections attributed to unprotected sex behavior among female sex workers (FSWs), transmission among HIV-serodiscordant couples, and transmission among men who have sex with men (MSM) [3]. HIV-serodiscordant couples, in which one partner is HIV-positive and the other is HIV-negative, are now recognized as a priority for HIV prevention interventions. The transmission risk for HIV-negative partners in HIV-serodiscordant couples may exceed 10% per year [4]. In sub-Saharan Africa, population surveys and mathematical models estimate that transmission within stable heterosexual serodiscordant relationships may account for more than 60% of new HIV infections [5], [6]. In China, a recent retrospective cohort study indicated that the HIV infection rate was 2.6 per 100 person-years (95%CI 2.4–2.8) among 14,805 treatment-naïve HIV-serodiscordant couples [7]. However, a cohort study conducted in Yining city of Xinjiang showed that HIV infection rate was as high as 32.5 per 100 person-years among 22 HIV-serodicordant couples, although such high incidence might be due to lack of random sampling and small sample size [8]. Therefore, prevention of HIV transmission among HIV-serodiscordant couples in Xinjiang is crucial to halting its spread among the general population. However, the current “ABC” approach of abstinence, being faithful, and condom use is only partially effective, highlighting the need for new and effective interventions.

Besides vaccination, research has focused on a variety of new prevention strategies such as post-exposure prophylaxis (PEP), microbicides, and pre-exposure prophylaxis (PrEP) [9]. Oral PrEP is a new HIV prevention approach in which people who are HIV-negative take oral anti-retrovirals (ARVs) to reduce the risk of becoming infected. Both current and planned oral PrEP efficacy trials were focused on two ARVs: tenofovir disoproxil fumarate (TDF) and Truvada which was a combination of TDF and emtricitabine (FTC). Efficacy and safety of oral PrEP have been tested among different at-risk populations such as MSM (iPrEx study) [10], heterosexual women and/or men (FEM-PrEP, TDF2, VOICE studies) [11], [12], [13], and HIV-serodiscordant couples (Partners PrEP) [14]. In addition, another study is ongoing to evaluate the efficacy and safety of TDF alone among injection drug users (Bangkok Tenofovir Study) [15]. Some of these studies yielded promising results. The iPrEx trial reported daily use of oral TDF/FTC reduced HIV infection among MSM by 44% [10]. The TDF2 trial found a once-daily use of TDF/FTC reduced the risk of acquiring HIV infection by roughly 62% overall in the study population of uninfected heterosexually-active men and women [12]. The Partners PrEP trial recently reported among 4758 serodiscordant couples from Kenya and Uganda, once daily use of oral TDF alone or TDF/FTC was associated with risk reduction of 67% and 75%, respectively, when provided with other HIV prevention services [14]. These findings suggest the safety and effectiveness of oral PrEP in HIV-serodiscordant couples. However, the FEM-PrEP trial, conducted by Family Health International in cooperation with research centers in Africa, was stopped early due to lack of efficacy of oral TDF/FTC in heterosexual women. Similarly, neither oral TDF nor oral TDF/FTC demonstrated efficacy in the VOICE study [16], [17]. These conflicting findings indicate the need to better understand factors influencing the effectiveness of oral PrEP (e.g., willingness to take PrEP, suboptimal adherence, and risk compensation).

Awareness of and willingness to use oral PrEP are important factors to consider when recommending this approach. However, willingness to use PrEP has been evaluated only among MSM and FSWs in China [18], [19]. Therefore, the aim of this study is to investigate awareness of and willingness to use oral PrEP among HIV-negative partners in HIV-serodiscordant couples in Xinjiang, China. In addition, we aim to identify factors that predict willingness to use oral PrEP to facilitate implementation of this prevention strategy.

## Materials and Methods

### Study design and participants

This study is a cross-sectional survey which was conducted between November 2009 and December 2010 in Urumqi (capital of Xinjiang province), Yining (located in the north of Xinjiang), and Kuche (located in the south of Xinjiang). Participants for this cross-sectional survey were recruited based on convenience sampling. Inclusion criteria were age 18 years or older, being sexual active, HIV-negative status, being married or cohabitation with HIV-positive partner ≥3 years, being heterosexual, ability to read and understand the questionnaire, and being willing to participate in this study and provide written informed consent. Those who were unaware of being in a HIV-serodiscordant relationship were not included in this study.

All individuals in China who test HIV-positive must be reported to the National Center for HIV/AIDS Control and Prevention at the Chinese Center for Disease Control and Prevention (China CDC). The local CDCs are responsible for following up the individuals who have reported a spouse or regular sex partner with whom they cohabit, and performing the detection of HIV for their HIV-negative partners every six month. The data on these individuals are recorded in the national HIV epidemiology database by both local and China CDC [7]. According to this database, staff members of local CDCs identified 382 HIV-negative partners in HIV-serodiscordant couples who were eligible for this study in Urumqi, Yining, and Kuche. These individuals were then informed by telephone of the study purpose and procedure, confidentiality parameters, and compensation for travelling expenses by local CDC staff members. They were encouraged to visit the local CDC office for an HIV test and to complete a self-administered questionnaire if they decided to participate in this study. Between November 2009 and December 2010, trained interviewers from Xinjiang Medical University conducted face-to-face interviews with participants who visited the local CDC office with the support of local CDC. After providing written informed consent, participants were asked to complete a self-administered questionnaire in a private room under the guidance of interviewers. In addition, blood specimens were collected for HIV test, and test results were shared with participants. The participants were compensated with a small gift worth 30 Renminbi (RMB, official currency of China) and travel expenses (10–20 RMB).

### Ethics statement

Chongqing Medical University Biomedical Research Ethics Committee and Xinjiang Medical University First Affiliated Hospital Ethics Committee reviewed and approved the study before implementation, and all participants provided written informed consent before taking part in the study. All private information from questionnaire were kept confidential and used for population analysis only.

### Data collection

Before receiving the questionnaire, all participants received brief explanations of oral PrEP and detailed descriptions of the usage, safety, and effectiveness of oral ARVs used as PrEP. They were encouraged to ask the interviewer to repeat any part that was unclear. In addition, terms in the explanation that were not understood by participants were further described with plain language. The explanation of oral PrEP was as follows:

“Oral pre-exposure prophylaxis (PrEP) is an HIV prevention approach that involves the use of medicines called antiretrovirals (ARVs) by people who are HIV-negative. The idea behind PrEP is that taking an ARV routinely may help prevent infection in people who come in contact with the virus, such as through unprotected sex. ARVs have been used successfully to treat millions of HIV-infected people worldwide. Because ARVs are effective in treating HIV, the hope is that they can prevent HIV infection as well. Currently, clinical trials are assessing the effectiveness and safety of oral PrEP in different at-risk populations, including HIV-negative partners in HIV-serodiscordant couples. At the present time, both current and planned trials of oral PrEP are focused on two ARVs approved for HIV treatment: tenofovir disoproxil fumarate (TDF) and Truvada. Truvada is a combination drug that contains TDF and emtricitabine (FTC). The suggested dosages are 300 mg once a day for TDF and 300 mg TDF/200 mg FTC once a day for Truvada. Both drugs have possible side effects similar to other ARV drugs, such as nausea, vomiting, and headache, but these side effects improve after a few weeks of oral PrEP use. Results of some trials suggest that oral PrEP could help reduce the risk of HIV infection in men who have sex with men and heterosexual women; however, the effectiveness of oral PrEP may be different in HIV-serodiscordant couples. Whether oral PrEP is safe and effective among Chinese HIV-serodiscordant couples is still unknown. Although there are some challenges in PrEP research, it is still a promising HIV prevention strategy. In this study, we invited you to answer questions about your awareness of oral PrEP, and we want to know if you are willing to use oral PrEP to prevent HIV or have concerns about its use if oral PrEP is proven to be both effective and safe in the Chinese population.”

After all participants understood the explanation of oral PrEP, they completed a self-administered questionnaire in a private room. Questionnaire applied in this study was designed by Chongqing Medical University study group, and questionnaire items were discussed with experience researchers and field workers to check pertinence and clarity of wording. The questionnaire contained items related to demographic characteristics (age, gender, place of residence, ethnicity, education level, employment status, and monthly household income), awareness of HIV/AIDS transmission routes (e.g., “Does HIV spread through dining with HIV-infected individual?”, “Does HIV spread through mosquito bites?”, “Does HIV spread through saliva, tears or sweats?”, etc.), awareness of HIV/AIDS prevention methods (e.g., “Can HIV/AIDS be prevented by consistent and correct use of high-quality condoms?”, etc.), and attitudes/behaviors related to HIV/AIDS (e.g., “Are you concerned that HIV may pose a threat to your family?”, “Are you planning to have children?”, “Do you agree it is difficult to prevent HIV/AIDS when cohabiting with a HIV-positive partner”, “Did you have sex with your HIV-positive partner in previous 6 months?”, etc.). Participants were also asked about awareness of, use of, and concerns about oral PrEP; acceptability of oral PrEP in terms of cost, adherence, and accessibility; and perceived behavioral changes after oral PrEP use. Willingness to use oral PrEP was surveyed with the following question “If oral PrEP was proven to be both safe and effective, would you be willing to use it for HIV prevention”. Participants were asked to report their intention on 5-point scale: 1 (I am definitely willing to use it) to 5 (I am definitely not willing to use it). To provide a conservative estimate of the intention to use oral PrEP, data were dichotomized into “willing to use oral PrEP” (score of 1 or 2) and “not willing to use oral PrEP” (3 or higher).

### Laboratory tests

To determine HIV status, blood specimens were first assessed by enzyme-linked immunosorbent-assay (Beijing KingHawk Pharmaceutical Co. Ltd., China). Positive results were confirmed by HIV-1/2 Western blot analysis (MP Biomedical Co. Ltd., Singapore).

### Statistical analysis

Questionnaire data were double entered, managed with EpiData 3.0 (Epidata Association, Odense, Denmark), and analyzed with SPSS (PAWS Statistics18). Descriptive statistics were used to assess factors such as demographic characteristics, awareness of HIV/AIDS transmission and prevention, attitudes/behavior related to HIV/AIDS, awareness of oral PrEP, and willingness to use oral PrEP. Univariate logistic regression analysis was used first to evaluate the relationship between willingness to use oral PrEP and demographic characteristics, behaviors/attitudes related to HIV/AIDS, attitudes toward oral PrEP use, and previous use of oral PrEP. Multivariate logistic regression analysis was then used to identify independent predictors of willingness to use PrEP. Factors that were significant at *P*<0.2 in univariate logistic regression analysis were entered into the initial multivariate logistic regression model. Using a backward stepwise procedure, factors that were not significant in the initial model were excluded to construct the final multivariate model. Significance was set at *P*<0.05. Odds ratios (ORs) were reported with 95% confidence intervals (CIs).

## Results

### Demographic characteristics

Among 382 HIV-negative partners in HIV-serodiscordant couples, 23 refused to participate in this study and eight were found to be HIV-positive; As a result, we obtained 351 questionnaires (response rate = 91.9%). Mean participant age was 34.8 (standard deviation 7.23 years, range 19–69 years), 298 (84.9%) participants were female, and 297 (84.6%) were ethnic Uyghur. Regarding education, 229 (65.6%) participants had only junior high education or below. Over half (51.3%) reported being unemployed, and 263 (74.9%) had a monthly household income <1000 RMB (155 US Dollars). Participant demographics stratified by willingness to use oral PrEP are shown in Table 1. Results of univariate logistic regression analysis showed that “monthly household income” was associated with willingness to use oral PrEP (Table 1).

**Table 1:** Relationship between demographic characteristics and willingness to use oral PrEP.

		Willing to use oral PrEP			
Factors	N (%)	Yes, n (%)	No, n (%)	OR (95% CI)	*P* value
Place of residence					0.76
Urumqi	141 (40.2)	117 (83.0)	24 (17.0)	0.86 (0.43–1.74)	0.68
Yining	110 (31.3)	95 (86.4)	15 (13.6)	1.12 (0.52–2.42)	0.78
Kuche	100 (28.5)	85 (85.0)	15 (15.0)	1.00	
Gender					0.73
Male	53 (15.1)	44 (83.0)	9 (17.0)	0.87 (0.40–1.91)	
Female	298 (84.9)	253 (84.9)	45 (15.1)	1.00	
Ethnicity					0.17
Uyghur	297 (84.6)	253 (85.2)	44 (14.8)	1.30 (0.51–3.33)	0.58
Han	20 (5.7)	14 (70.0)	6 (30.0)	0.50 (0.14–1.84)	0.30
Other	34 (9.7)	28 (82.4)	6 (17.6)	1.00	
Age					0.35
<35 years	215 (61.3)	185 (86.0)	30 (14.0)	1.32 (0.74–2.37)	
≥35 years	136 (38.7)	112 (82.4)	24 (17.6)	1.00	
Education					0.85
Elementary school	108 (30.9)	92 (85.2)	16 (14.8)	1.15 (0.56–2.35)	0.70
Junior high school	121(34.7)	104 (86.0)	17 (14.0)	1.22 (0.61–2.47)	0.57
Senior high school and beyond	120 (34.4)	100 (83.3)	20 (16.7)	1.00	
Employment status					0.43
Employed	170 (48.7)	141 (82.9)	29 (17.1)	0.79 (0.44–1.41)	
Unemployed	179 (51.3)	154 (86.0)	25 (14.0)	1.00	
Monthly household income					0.01
<1000 RMB	263 (74.9)	230 (87.1)	33 (12.9)	2.19(1.19–4.03)	
≥1000 RMB	88 (25.1)	67 (75.0)	21 (25.0)	1.00	

Note: A total of 349 participants provided responses for employment status and education.

Abbreviations: PrEP, pre-exposure prophylaxis; CI, confidence interval; OR, odds ratio; RMB, Renminbi.

### Awareness of HIV/AIDS transmission and prevention

This section of questionnaire contained 11 questions about HIV/AIDS transmission routes and prevention methods (Table 2). More than 96% of the participants correctly responded that HIV could be transmitted by blood transfusion, needle sharing injection drug use, and mother to child transmission during pregnancy, birth, or breast-feeding. However, 125 (35.6%) incorrectly believed HIV could not be transmitted by unprotected sex with a clean and healthy-looking HIV-infected individual, 66 (18.8%) believed that HIV could be transmitted by mosquito bites, and 47 (13.4%) believed that HIV could be transmitted by saliva, tears, or sweats. Regarding HIV/AIDS prevention, 175 (49.9%) believed that washing genitals before and after sex could prevent HIV/AIDS.

**Table 2:** Awareness of HIV transmission routes and prevention methods (n = 351).

Questionnaire items	Correct answers (n)	Awareness rate (%)
Can HIV/AIDS be transmitted by: (correct answer)		
Blood transfusion? (yes)	340	96.9
Needle-sharing injection drug use? (yes)	338	96.3
From mother to child during pregnancy, birth, or breast-feeding? (yes)	341	97.2
Dining with HIV-infected individual? (no)	329	93.7
Mosquito bites? (no)	285	81.2
Unprotected sex with clean, healthy-looking HIV-infected individual? (yes)	226	64.4
Saliva, tears, or sweats? (no)	304	86.6
Can HIV/AIDS be prevented by: (correct answer)		
Consistent and correct use of high-quality condoms? (yes)	329	93.7
Being in a long-term mutually monogamous relationship with an uninfected partner? (yes)	281	80.1
Using disposable needles without sharing? (yes)	309	88.0
Washing genitals before and after sex? (no)	176	50.1

Note: Awareness rate  =  number of participants with correct answers/total participants×100%.

Abbreviations: HIV, human immunodeficiency virus; AIDS, acquired immune deficiency syndrome.

To assess the relationship between levels of participants' awareness of HIV/AIDS and their willingness to use oral PrEP, we classified our participants into “having a good awareness of HIV/AIDS” group and “not having a good awareness of HIV/AIDS” group based on the number of questions each participant correctly answered. We set the scale cut-off value as “10 questions” to allow for two groups of similar size. Thus, 167 (47.6%) participants correctly answered at least 10 questions or had a good awareness of HIV/AIDS. Results of univariate logistic regression analysis showed “having a good awareness of HIV/AID” was not associated with willingness to use oral PrEP (*P* = 0.09).

### Behaviors and attitudes related to HIV/AIDS

Regarding behaviors and attitudes related to HIV/AIDS, 295 (84.0%) participants reported having sex with an HIV-positive partner in the previous 6 months; of these participants 236 (80.0%) reported condom use whenever having sex, and 59 (20.0%) reported having unprotected sex with the HIV-positive partner (Table 3). In addition, 61 (17.4%) participants reported they were planning to have children. In this section of questionnaire, participants were asked, “Do you agree that it is difficult to prevent HIV/AIDS when cohabiting with a HIV-positive partner?”, and answers were 5-point scale: 1 (Yes, totally agree) to 5 (No, totally disagree), and data were dichotomized into “Yes” (score of 1 or 2) and “No” (3 or higher). As a result, 118 (33.6%) agreed that it was difficult to prevent HIV/AIDS when cohabiting with a HIV-positive partner. Participant's perception of HIV risk was surveyed with the following question “Do you perceive yourself as likely to contract HIV from your HIV-positive partner” (1 =  “yes, very likely”; 2 =  “yes, somewhat likely”; 3 =  “no, somewhat unlikely”; 4 =  “no, very unlikely”), and data were dichotomized into “Likely” (score of 1 or 2) and “unlikely” (3 or 4). As a result, 109 (31.1%) participants perceived themselves as likely to contract HIV from their HIV-positive partners. Results of univariate logistic regression analysis showed that “it is difficult to prevent HIV/AIDS when cohabiting with a HIV-positive partner” and “self-perceived likelihood of contracting HIV from HIV-positive partner” were associated with willingness to use oral PrEP (Table 3).

**Table 3:** Relationship between behaviors/attitudes related to HIV/AIDS and willingness to use oral PrEP.

		Willing to use oral PrEP				
Factors	N (%)	Yes, n (%)		No, n (%)	OR (95% CI)	*P* value
It is difficult to prevent HIV/AIDS when cohabiting with a HIV-positive partner						0.03
Yes	118 (33.6)	107 (90.7)		11 (9.3)	2.20 (1.09–4.45)	
No	233 (66.4)	190 (81.5)		43 (18.5)	1.00	
Planning to conceive children						0.53
Yes	61 (17.4)	50 (82.0)		11 (18.0)	0.79(0.38–1.64)	
No	290 (82.6)	247 (85.2)		43 (14.8)	1.00	
Being concerned HIV may pose a threat to family members						0.41
Yes	268 (76.8)	229 (85.4)		39 (14.6)	1.32(0.68–2.53)	
No	82 (23.2)	67 (81.7)		15 (18.3)	1.00	
Sex with HIV-positive partner in previous 6 months						0.18
Yes	295 (84.0)	253 (85.8)		42 (14.2)	1.64(0.80–3.37)	
No	56 (16.0)	44 (78.6)		12 (21.4)	1.00	
Frequency of condom use in previous 6 months						0.48
Every time	236 (80.0)	201 (85.2)		35 (14.8)	1.00	
Most of the time	21 (7.1)	20 (95.2)		1 (4.8)	3.48(0.45–26.79)	0.23
Sometimes	13 (4.4)	12 (92.3)		1 (7.7)	2.09(0.26–16.58)	0.49
Never	25 (8.5)	20 (80.0)		5 (20.0)	0.70 (0.25–1.98)	0.50
Self-perceived likelihood of contracting HIV from HIV-positive partner						0.01
Likely	109 (31.1)		101 (92.7)	8 (7.3)	2.96 (1.35–6.52)	
Unlikely	242 (68.9)		196 (81.0)	46 (19.0)	1.00	

Abbreviations: HIV, human immunodeficiency virus; AIDS, acquired immune deficiency syndrome; PrEP, pre-exposure prophylaxis; CI, confidence interval; OR, odds ratio.

### Awareness of, use of, and attitudes toward PrEP

After receiving an explanation of oral PrEP, 341 (97.2%) participants reported they had never heard of it before, 7 (2.0%) reported having taken medicine to prevent sexually transmitted diseases, 2 (0.6%) reported having taken PrEP to prevent HIV transmission, and 8 (2.3%) heard of others who had taken PrEP (Table 4). In addition, 147 (41.8%) participants believed that PrEP might be effective, and 317 (90.3%) believed that PrEP should be available to a larger population if proven to be effective and safe. Stigma associated with oral PrEP use was assessed by single item question “Do you worry about being discriminated against by others if you use oral PrEP for HIV/AIDS prevention” (1 =  “yes, definitely”; 2 =  “yes, probably”; 3 =  “no, probably not”; 4 =  “no, definitely not”), and again data were dichotomized into “Yes” (score of 1 or 2) and “No” (3 or 4). Results of univariate logistic regression analysis showed “worrying about being discriminated against by others due to oral PrEP use” was associated with willingness to use oral PrEP, suggesting participants who feared of stigma due to oral PrEP use had lower odds of being willing to use oral PrEP (Table 4).

**Table 4:** Relationship between awareness of/use of/attitudes toward PrEP and willingness to use PrEP.

		Willing to use oral PrEP			
Factors	N (%)	Yes, n (%)	No, n, (%)	OR (95% CI)	*P* value
Ever heard of vaginal microbicides					0.11
Yes	27 (7.7)	26 (96.3)	1 (3.7)	5.09 (0.68–38.29)	
No	324 (92.3)	271 (83.6)	53 (16.4)	1.00	
Ever heard of PEP					0.70
Yes	34 (9.7)	28 (82.4)	6 (17.6)	0.83 (0.33–2.12)	
No	317 (90.3)	269 (84.9)	48 (15.1)	1.00	
Ever heard of PrEP					0.68
Yes	10 (2.8)	8 (80.0)	2 (20.0)	0.72(0.15–3.49)	
No	341 (97.2)	289 (84.8)	52 (15.2)	1.00	
Ever taken medicine to prevent sexually transmitted disease					0.94
Yes	7 (2.0)	6 (85.7)	1 (14.3)	1.09 (0.13–9.26)	
No	344 (98.0)	291 (84.6)	53 (15.4)	1.00	
Worrying about being discriminated against by others due to oral PrEP use					0.00
Yes	192 (54.7)	145 (75.5)	47 (24.5)	1.00	
No	159 (45.3)	152 (95.6)	7 (4.4)	7.04 (3.08–16.67)	

Abbreviations: PEP, post-exposure prophylaxis; PrEP, pre-exposure prophylaxis; CI, confidence interval; OR, odds ratio.

### Willingness to use oral PrEP and concerns related to its use

A total of 297 participants (84.6%) were willing to use oral PrEP if proven both effective and safe. The remaining 54 participants (15.4%) were unwilling to use oral PrEP because 31 (57.4%) believed they were at no risk of contracting HIV, or 28 (51.9%) were concerned about its safety, or 12 (22.2%) doubted its efficacy.

Among participants willing to use oral PrEP, 258 (86.8%) were concerned about its efficacy, 249 (83.8%) were concerned about its safety, 190 (64.0%) were concerned about its cost, and 45 were concerned about its availability (15.2%). Regarding social concerns, 113 (38.0%) participants had no fear of disclosing their use of PrEP to others.

### Acceptability of oral PrEP in terms of cost and accessibility

Among participants willing to use oral PrEP, 118 (39.7%) believed oral PrEP should be provided at no cost, 106 (35.7%) responded that they could afford to pay up to 100 RMB (approximately 14 US Dollars), 38 (12.8%) could afford to pay 100 to 200 RMB (14–28 US Dollars), 35 (11.8%) could afford to pay more than 200 RMB (28 US Dollars).

Among participants willing to use oral PrEP, 198 (66.7%) preferred it to be available at local CDC offices, 95 (32.0%) preferred it to be available at voluntary counseling and testing centers, and 70 (23.7%) preferred it to be available at hospitals.

### Perceived behavioral changes after oral PrEP use

Among participants willing to use oral PrEP, 262 (88.2%) reported they would not decrease their frequency of condom use if using oral PrEP and 287 (96.6%) reported they would not increase their number of sex partners.

### Multivariate logistic regression analysis of factors associated with willingness to use oral PrEP

In multivariate logistic regression analysis, willingness to use oral PrEP was coded as “1”, and unwillingness to use oral PrEP was coded as “0”. Variables that were significant (*P*<0.2) in the univariate analysis were entered into the initial multivariate logistic model; these variables included “age”, “ethnicity”, “monthly household income”, “having a good awareness of HIV/AIDS”, “sex with HIV-positive partner in the previous 6 months”, “awareness of vaginal microbicides”, “it is difficult to prevent HIV/AIDS when cohabiting with a HIV-positive partner”, “self-perceived likelihood of contracting HIV from an HIV-positive partner”, and “worrying about being discriminated against by others due to oral PrEP use”. In the final multivariate logistic regression model (Table 5), independent factors predicting willingness to use oral PrEP were “monthly household income” (adjusted OR = 2.78, <1000 RMB vs. ≥1000 RMB, 95% CI: 1.36–5.69), “perceived likelihood of contracting HIV from HIV-positive partner” (adjusted OR = 2.63, likely vs. unlikely, 95% CI: 1.12–6.19), and “worrying about being discriminated against by others for using PrEP” (OR = 9.43, no vs. yes., 95% CI: 3.78–23.50).

**Table 5:** Fitted multivariable logistic regression model for predicting willingness to use oral PrEP.

Factors	Adjusted OR	95% CI	*P* value
Monthly household income			
<1000 RMB	2.78	1.36–5.69	0.01
≥1000 RMB	1.00		
Self-perceived likelihood of contracting HIV from HIV-positive partner			
Likely	2.63	1.12–6.19	0.03
Unlikely	1.00		
Worrying about being discriminated against by others due to oral PrEP use			
No	9.43	3.78–23.50	<0.001
Yes	1.00		

Abbreviations: PrEP, pre-exposure prophylaxis; CI, confidence interval; OR, odds ratio.

## Discussion

To our knowledge, this is the first study to report the awareness of and willingness to use oral PrEP among HIV-negative partners in HIV-serodiscordant couples in China. We found that awareness of oral PrEP among HIV-negative partners in HIV-serodiscordant couples was only 2.8%, which was lower than that of MSM (11.2%) and FSWs (16.5%) in China [18], [19]. However, 84.6% of participants in this study were willing to use oral PrEP for HIV prevention if oral PrEP was proven to be both safe and effective. This rate was higher than that of MSM (67.8%) and FSW (69%) in China and that of MSM in the United States (67%–74.4%) [20], [21], [22], but was lower than that of serodiscordant couples in Kenya (92.7%) [23]; These findings suggest high acceptability of oral PrEP among HIV-negative partners in HIV-serodiscordant couples in China.

In this study, safety and effectiveness of oral PrEP were primary concerns of participants who were willing to use oral PrEP, as well as those who were not willing to. Although some studies have reported that oral PrEP is effective among MSM, FSWs, and serodiscordant couples [24], there are many unresolved issues that need further investigation (e.g., optimal drug combination, dosing interval, duration of oral PrEP, HIV testing frequency, safety monitoring, and strategy for PrEP discontinuation) [25]. In addition, these studies also reported the potential side effects of oral PrEP such as kidney damage [10], liver damage [11], and reduction in bone density [12]. Nowadays, patients are seeking greater engagement in health care choices, increasing the demand for high-quality information about clinical options [26]. Similarly, to make a balanced decision, potential oral PrEP users may require unbiased information on both the benefits and harms of oral PrEP. Therefore, detailed effectiveness and safety profile of oral PrEP should be given prior to PrEP initiation in order to maximize informed decision-making among potential users.

Willingness to use PrEP may depend on some factors such as perception of HIV risk and fear of social stigma [27]. In our study, participants who perceived themselves as likely to contract HIV from their partners were more likely to be willing to use oral PrEP, whereas those who worried about being discriminated against by others due to oral PrEP were less likely to be willing to use it. Similarly, these factors influenced the willingness to use condoms [28] and HIV vaccine [29]. Therefore, efforts should be made to reduce the stigma associated with oral PrEP use, to demonstrate and promote its benefits, and to increase confidence in their ability to effectively use oral PrEP.

We found cost might pose a barrier to oral PrEP use among HIV-negative partners in HIV-serodiscordant couples. 74.9% of participants had a monthly household income <1000 RMB (155 US Dollars), and they were more likely to be willing to use oral PrEP than participants who had household incomes ≥1000 RMB (adjusted OR = 2.78). However, most of them reported they could afford oral PrEP only if it cost <200 RMB per month (14 US Dollars). In 2003, the Chinese government launched a policy of “Four Frees One Care”, in which expensive ARVs are provided at no cost for all rural and urban poor people living with HIV. However, this policy doesn't cover uninfected high-risk populations [30]. Therefore, even though oral PrEP was proven to be effective and safe, it might be inaccessible to most of HIV-serodiscordant couples in China. In addition, consistent oral PrEP use may require frequent lab tests for HIV infection and safety monitoring, which will lead to extra expenses for potential users. Therefore, we suggest the government should take appropriate measures to reduce the price of ARV drugs so that oral PrEP is accessible to most of serodiscordant couples in China [31].

Oral PrEP is a promising approach to HIV prevention among HIV-serodiscordant couples, however, no single stand-alone prevention strategy is sufficient to curb the spread of HIV; thus, oral PrEP should be combined with other proven strategies such as antiretroviral therapy for prevention, voluntary medical male circumcision, behavioral intervention (e.g. condom use), etc [32].

The present study had several limitations worth noting. First, we used a convenience sample, which might result in selection bias and limit the generalization of our results. Second, the cross-sectional design based on a self-administered questionnaire may have introduced information bias such as recall bias. Finally, anticipated willingness does not always translate into actual behavior, and this willingness could change if other interventions to prevent HIV were available. In addition, whether HIV-positive partners of participants are on antiretroviral therapy, their CD4 cell level, and participants' awareness of antiretroviral therapy for prevention were not surveyed in this study, which may have influence on willingness to use oral PrEP.
